# Carotenoid-Chlorophyll Interactions in a Photosynthetic Antenna Protein: A Supramolecular QM/MM Approach

**DOI:** 10.3390/molecules23102589

**Published:** 2018-10-10

**Authors:** Matthew J. Guberman-Pfeffer, José A. Gascón

**Affiliations:** Department of Chemistry, University of Connecticut, Storrs, CT 06269-3060, USA; matthew.guberman-pfeffer@uconn.edu

**Keywords:** supramolecular QM/MM, PCP, peridinin, chlorophyll *a*, light harvesting

## Abstract

Multichromophoric interactions control the initial events of energy capture and transfer in the light harvesting peridinin-chlorophyll *a* protein (PCP) from marine algae dinoflagellates. Due to the van der Waals association of the carotenoid peridinin (Per) with chlorophyll *a* in a unique 4:1 stoichiometric ratio, supramolecular quantum mechanical/molecular mechanical (QM/MM) calculations are essential to accurately describe structure, spectroscopy, and electronic coupling. We show that, by enabling inter-chromophore electronic coupling, substantial effects arise in the nature of the transition dipole moment and the absorption spectrum. We further hypothesize that inter-protein domain Per-Per interactions are not negligible, and are needed to explain the experimental reconstruction features of the spectrum in wild-type PCP.

## 1. Introduction

The capture, conversion, and transfer of solar energy by artificial means constitute some of the greatest research challenges at present [1,2]. Natural architectures that overcome these difficulties have been evolved by photosynthetic algae, bacteria, and plants, which collectively consume an estimated five times more power than is needed for all human activities [3]. Lessons learned from the mechanistic study of natural light harvesting complexes will inform and inspire the rational design of artificial photosynthetic devices capable of meeting growing energy demands for sustainable human development [1,4,5].

**Figure 1:**
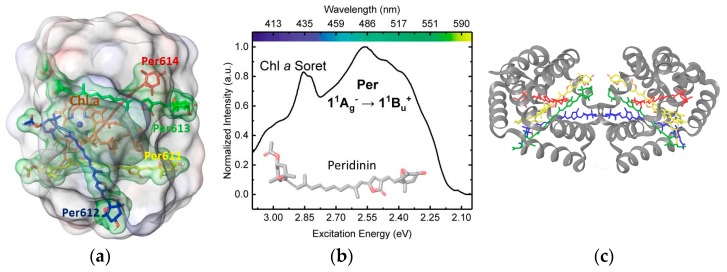
(**a**) Partial view of the cofactor cluster inside a single domain of the reconstituted, recombinant peridinin-chlorophyll *a* protein (RFPCP) complex. Highlighted in green is the supramolecular assembly consisting of four bound peridinin (Per) molecules and one chlorophyll *a* (Chl *a*) encapsulated by the van der Waals surface of the protein. (**b**) Absorption spectrum of the peridinin-chlorophyll *a* protein (PCP) complex (ref. [4]) and chemical structure of Per. (**c**) Full view of the RFPCP C_2_-symmetric homodimer.

A notable example of a highly efficient light harvesting antenna is the water-soluble peridinin-chlorophyll *a* protein (PCP) from marine algae dinoflagellates [6]. In the main form of PCP (MFPCP), isolated from *Amphidinium carterae* [7], the complex is characterized by a C_2_-pseudosymmetric, two domain α-helical protein capsule of densely packed chromophoric assemblies. Each cofactor cluster has a unique 4:1 stoichiometric ratio of the structurally distinctive carotenoid peridinin (Per) and chlorophyll *a* (Chl *a*), as seen in Figure 1a, as well as a molecule of the lipid digalactosyldiacylglycerol. Per is a heptene-based chromophore that features a C_37_, instead of the standard C_40_ carotenoid framework, as well as an unusual pairing of allenic and γ-butenolide moieties, as seen in Figure 1b, which significantly shapes the photophysical response of the polyene chain to the polarity, proticity, and polarizability of the local environment [6].

A reconstituted, recombinant PCP (RFPCP) complex (Figure 1c), which is a homodimer of the *N*-terminal domain from the wild-type protein, has been used as a model system to study both spectral tuning and excitation energy transfer [4]. The cofactor clusters in either MF- or RFPCP harvest blue-green photons with ~90% efficiency [4] by mechanisms intimately related to the structure of Per and the architecture of the solvated PCP complex [8].

The linear absorption spectrum of Per is commonly interpreted using a three-state model composed of the ground (S_0_) and first two excited singlet states (S_1_ and S_2_). In analogy to a rigorously C_2h_-symmetric polyene, the ground and first excited states are of “A_g_^−^” symmetry (quotes denote “approximate”), and one-photon transitions between these states are forbidden by symmetry selection rules [9]. Though the substitution pattern of the polyene chain in Per strongly breaks C_2h_ molecular symmetry, the S_0_ → S_1_ transition is still not observed experimentally. Fiedor et al. [10] have proposed an alternate explanation for the forbidden character of this transition which involves an unfavorable Frank-Condon overlap of the S_0_ and S_1_ potential energy surfaces due to severe geometric distortions in the S_1_ state. The second excited state has “B_u_^+^” symmetry. The S_0_ (“1^1^A_g_^−^“) → S_2_ (“1^1^B_u_^+^”) excitation is strongly one-photon allowed, dominates the optical spectrum in the 450–550 nm region, and represents the initial step of light harvesting by PCP. Once energy is collected by the Per spectral forms, it is transferred to the Qx and Qy states of Chl *a*, and ultimately shuttled into Photosystem II. The Qx and Qy states (600–700 nm) are the two lowest-lying excited states of Chl *a*, with Qx being the closest in energy to the most red-shifted absorber among the Pers (~510 nm). Qy is dominated by HOMO → LUMO and (HOMO − 1) → (LUMO + 1) configurations, whereas Qx is primarily (HOMO − 1) → LUMO and HOMO → (LUMO + 1) in character.

The S_2_ state of the four Pers within a functional unit of PCP are tuned to absorb at specific wavelengths distributed over a 58 nm range of maximal solar irradiance [4]. By contrast, the maximal solvatochromic shift reported for the S_2_ absorption maximum in a variety of homogeneous solvents is only 16–28 nm [6]. We recently reported multichromophoric quantum mechanical/molecular mechanical (QM/MM) simulations that delineated the controlling factors for wavelength regulation in PCP, and permitted the first unambiguous assignment of the distinct Per spectral signatures to specific chromophores [8]. Protein-induced conformational distortion, inter-chromophore electronic coupling, and the electrostatic environment of the complex, mediated by an aqueous dielectric, were found to make nearly equal and additive contributions to the S_0_ → S_2_ wavelength variation. These supramolecular QM/MM simulations also identified molecular excitons delocalized over two or three chromophores depending on the absorption wavelength, thereby directly addressing a long-standing debate over the presence and relevance of exciton delocalization on PCP optical properties [11,12,13].

Recent theoretical [14,15] and experimental [15,16,17] work has identified an ultrafast quantum coherent mechanism in the Per-to-Chl *a* energy transduction process. While it is now well established that Per-to-Chl *a* energy transfer represents a fundamental event in light harvesting by PCP, it is still unknown if energy transfer paths among Pers, and from multiple Pers to Chl *a*, help funnel the transfer of energy. A step towards such understanding would at least require a full characterization of electronic coupling strengths between all chromophoric cofactors. It is only recently, with the aid of supramolecular QM/MM approaches [8], that such evaluations can be accurately obtained. Supramolecular QM/MM refers to the implementation of QM/MM methods where the quantum mechanical (QM) regions involve entire supramolecular assemblies of distinct units. These units exhibit subtle interactions controlling structure, function and spectroscopy. In this report, we characterize the excitonic interactions in PCP to describe the strengths and environmental regulation of inter-chromophore electronic coupling. Our findings add to the detailed understanding of light harvesting strategies operative in this exceptionally efficient protein antenna complex.

## 2. Materials and Methods

### 2.1. Model Preparation

A model of the reconstituted PCP complex from the highest resolution crystal structure available (PDB ID 3IIS; 1.4 Å) [4] was prepared. The A and M chains, which non-covalently associate to form a C_2_-symmetric homodimer of the wild-type PCP *N*-terminal domain, were retained. Because of the symmetry, preparation decisions described for one subunit apply to both domains. Unless otherwise noted, QM regions were applied on the M chain pigments in the presence of the full homodimer. The reconstituted complex strongly resembles structurally and spectroscopically wild-type PCP [4,18]. Experimental spectral reconstructions for both complexes found four 0-0 bands that agree within 8 nm [4,18]. Three of these features correspond to absorption by two Pers that are presumably related by the C_2_ symmetry of the complex, whereas the fourth spectral origin is attributed to a single Per. A fifth spectral origin due to a single Per is only observed for the wild-type complex. This experimental data strongly suggests that an analysis of reconstituted PCP is transferrable to the wild-type complex, and applies to the spectral tuning of all but one Per in wild-type PCP.

Hydrogen atoms were added, protonation and tautomeric states assigned, and the H-bonding network optimized by reorienting donor/acceptor side chains, using the Protein Preparation application in Maestro version 10.6.014 [19]. Each chain contains 20 acidic and 18 basic residues (not counting His) that are primarily surface exposed, and these residues were assigned standard protonation states for pH 7.0. His-66 was assigned the neutral N tautomeric state, because the other imidazole nitrogen accepts a H-bond from a water molecule coordinated to Chl *a*. His-67 was assumed protonated (His-H+) following the assignment of Bricker and Lo [14]. For an optimized H-bonding network, the side chains of Asn-24, Gln-30, and Asn-102 were flipped by 180°. All bulk crystallographic waters, which constitute an incomplete and statistically unrepresentative configuration of the solvated state, were removed. To approximate the native environment for the water-soluble complex, an aqueous implicit solvent was modeled with the Poisson-Boltzmann finite difference (PBF) method [20] using the highest resolution grids available. However, a few crystallographic waters were retained, including the water molecule that serves as the fifth coordination to the Mg of Chl *a*, and a structural water that participates in a bridging H-bonding network between Tyr-136 and Per613. The solvent accessible surface in the PBF calculations was formed by considering the full homodimer.

### 2.2. Structure Optimizations and QM/MM Simulations

The four Pers and Chl *a* of a cofactor cluster were subjected to torsionally restrained optimizations in the respective binding sites to assess the relevance of experimental coordinate uncertainty. Dihedrals are generally regarded as more reliable from X-ray crystallographic structures than bond lengths and angles [21]. Excitation energies changed by <60 meV upon optimization for all four Pers and Chl *a* when individually included in the QM region, and therefore we chose to use the X-ray (non-optimized) geometries in all the single or multichromophore QM/MM calculations.

We have shown before that the absorption wavelengths agreed within 5 nm with or without Chl *a* added to the QM region, and that the nature of the Per S_0_ → S_2_ transitions were unaffected. Therefore, the QM region in all the supramolecular QM/MM calculations of this current work only contains Per molecules (up to eight Per molecules in the largest set-up).

All QM/MM simulations, except for the calculation of PBF charges, were performed in Gaussian 16 Rev. B.01 (Gaussian Inc, Wallingford, CT, USA), [22]. The B3LYP/6-31g(d) model chemistry and the Amber99 force field were used for the QM and MM regions, respectively. This methodology was used to calculate electrostatic potential surface (ESP) charges on all non-standard residues, as well as to perform the restrained optimizations. The MM region was converted to a background charge distribution for the inter-pigment electronic interaction analysis described in a following subsection. This step was necessary because the electronic energy transfer (EET) methodology [23,24] in Gaussian 16 uses fragment definitions that currently conflict with the layer specifications needed for QM/MM calculations using the ONIOM method. PBF charges were added to the background charge distribution for excitation calculations to model the effect of solvent on inter-chromophore interactions. PBF charges were obtained from a single point calculation on the RFPCP complex in Qsite [25] from Schrodinger LLC. (New York, NY, USA), in which all four Pers within a cofactor cluster of the complex were included in the QM region. These equilibrated PBF charges were used to represent the solvent reaction field in the Gaussian QM calculations as part of the background charge distribution. The PBF charges were calculated using the B3LYP/LACVP * model chemistry and the OPLS-AA force field for the QM and MM regions, respectively. This Qsite calculation was performed with the fully analytical self-consistent field (SCF) method, the highest resolution integration grids available for density functional theory (DFT), and with the default of level-shifting virtual orbitals disabled. The transferability of the PBF charges from Qsite to Gaussian was tested by comparing the results of vertical excitation calculations in both programs. In one case, vertical excitation energies were calculated with Qsite (B3LYP/LACVP*/OPLS/PFB) and in the other case with ONIOM (B3LYP/LACVP*/Amber/PFB charges). Notwithstanding differences in the level of theory and types of force fields used in the Gaussian and Qsite calculations, excitation energies and oscillator strengths agreed within ~2 nm and 0.5 units, respectively.

### 2.3. Excited States and Electronic Coupling Analysis

Vertical excitations were computed at the time dependent DFT (TD-DFT) level with the Tamm-Dancoff approximation (TDA). This methodology has been successfully applied previously to predict carotenoid spectra [26]. We were exclusively interested in predicting the transition to the “1^1^B_u_^+^” state, which is dominated by single excitation configurations [27]. TD-DFT/TDA is expected to perform well for transitions of this nature, and we consistently reproduced experimental absorption maxima within 0.11 eV, which is well within the accepted error for the method [28].

Inter-pigment electronic interactions were investigated through a supramolecular QM/MM or QM/background electrostatics approach, and the electronic energy transfer methodology implemented in Gaussian 16, which will be referred to hereafter as EET. The EET method [23,24] is a perturbative scheme that calculates the electronic interaction between the transition densities of fragment regions as an approximation to the full QM calculation of the multichromophoric system.

EET was used to compute coupling constants between the Per and Chl *a* chromophores of PCP at the TDA-B3LYP/6-31g(d) level of theory. The eight Pers and two Chl *a* molecules in RFPCP were each defined as a fragment for the EET analysis. To perform these calculations in the presence of the PCP complex (protein and lipid molecules) with or without an implicit aqueous solvent, Amber partial atomic charges for the protein and/or PBF charges from the Qsite calculation were added as a background charge distribution. S_2_ excitation wavelengths were obtained from this approach by constructing and diagonalizing a Hamiltonian of the chromophore site energies and coupling constants. These wavelengths were directly compared to the wavelengths from the full supramolecular QM calculation that included the Amber and PBF charges as a background electrostatic distribution. Section 3.1 shows the validity of this approach.

In a further calculation that examined the effect of coupling among the Pers on their interaction strength with Chl *a*, the four Pers in each domain of RFPCP were defined as a single fragment which interacted with the Chl *a* molecules of the complex. Each Chl *a* in the two domains was considered a separate fragment.

### 2.4. Natural Transition Orbital (NTO) Analysis

The nature of the computed excitations was inspected using natural transition orbitals [29,30]. Predicted absorptions for the pigment assembly were assigned to the chromophore with an NTO hole-particle pair that represented at least 50% of the character of the excitation. Excitonic states and transition dipole moments for the supramolecular QM/MM calculations will be referred to by these single-chromophore assignments, even though the excitations are delocalized over multiple pigments.

## 3. Results

### 3.1. On the Accuracy of Excitonic Coupling Models

The most accurate description of an electronically interacting assembly of chromophores would, in principle, involve a large-scale QM or QM/MM calculation. While we have carried out such calculations on a single domain of PCP, this approach becomes intractable when considering chromophore interactions between the two protein domains at the TD-DFT level. Here, however, we show that direct calculation of electronic couplings via EET, followed by diagonalization of the exciton Hamiltonian for the four Pers within a single PCP domain gives comparable results to a large-scale calculation in which the QM region contains all four Pers. We find that the excitonic model reproduces well the more accurate supramolecular QM/MM absorption wavelengths with a maximum difference of 2–12 nm as seen in Table 1. The largest discrepancy occurs under electronic embedding, perhaps reflecting the different polarization responses of four separate versus one large QM region. The overall close agreement of the two approaches attests to the validity of the less computationally demanding fragment-based excitonic model. Thus, we propose that the EET approach can be used to obtain excitation energies for supramolecular assemblies that are too large to be described with a direct QM calculation.

**Figure 2:**
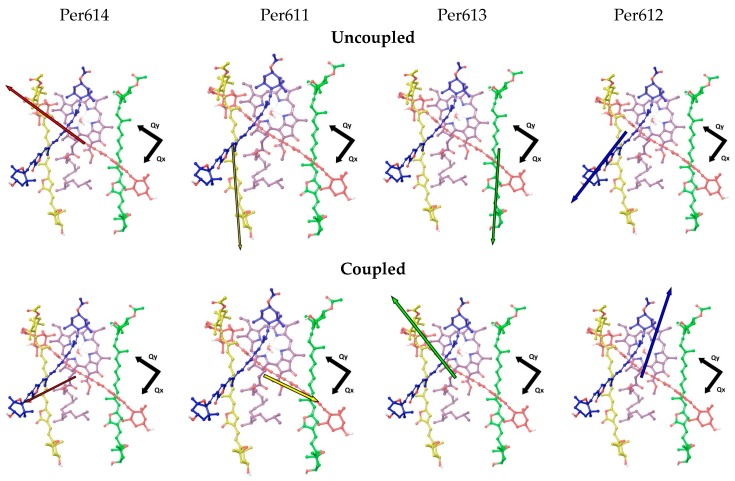
Visualization of the S_0_ → S_2_ TDMs for (top panel) uncoupled and (bottom panel) coupled Pers in a functional domain of PCP. The coupled states are assigned to the Per that carries at least 50% of the excitation, and the associated TDM vector is colored to match the given Per. The black vectors point in the directions of the Qy and Qx TDMs of the uncoupled Chl *a*, which are shown in each part of the figure to aid a comparison of the angle between the Per and Chl *a* TDMs. All TDMs were calculated in the presence of the aqueously solvated PCP complex.

**Figure 3:**
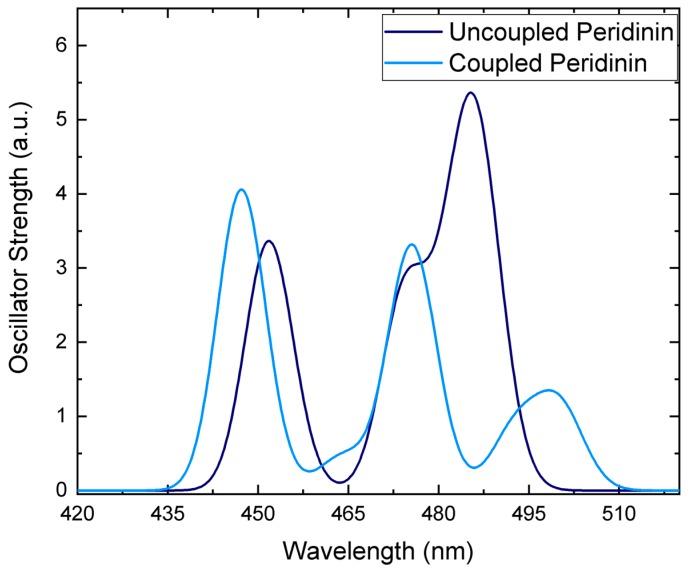
Simulated absorption spectra for the uncoupled (dark blue) and coupled (light blue) four Pers of a cofactor cluster in the solvated PCP complex. Gaussians with a full width at half maximum of 8 nm were fit to the calculated excitation wavelengths.

The supramolecular QM/MM calculations also show that the inter-chromophore interactions change the magnitude and direction of the S_2_ transition dipole moments (TDMs; Figure 2), which is reflected in a redistribution of oscillator strength to shorter-wavelength absorption bands as seen in Figure 3. Chromophore coupling also red shifts the lowest-lying Per excitons, thereby helping to close the energetic gap between the Per and Chl *a* excited states. The EET analysis complements these findings by quantifying the pairwise inter-chromophore interactions that are responsible for the wavelength regulation and oscillator strength redistribution predicted in the supramolecular simulation.

When chromophore electronic coupling is neglected, as shown in the top panel of Figure 2, the TDMs of Per614 and Per612 are nearly aligned to the Chl *a* Qy and Qx TDMs, respectively. The TDMs of Per611 and Per613 are oriented 37–43° with respect to the Qy TDM. All the Per TDMs have magnitudes of 17–18 D. When chromophore electronic coupling is introduced among all the Pers in a cofactor cluster, the TDMs for the excitons predominately carried by Per614 and Per612 are 41° and 28° respectively from the Qx TDM of Chl *a*. The TDMs for the Per611- and Per613-centered excitations are 20–29° from the Qy TDM of Chl *a*.

The TDM re-orientations induced by chromophore coupling are seemingly consistent with the previous finding that dimeric Per excitons enhance energy transfer to Chl *a* relative to monomeric Per excited states [14]. However, the magnitudes of the TDMs are also substantially altered through exciton delocalization. The TDMs for the Per611- and Per614-centered excitations are decreased by 6.9–7.9 D, whereas the TDMs for the Per612- and Per613-centered excitons are increased by 1.7 D. The close proximity of the chromophores further argues against a simple dipole-dipole interaction analysis. Consideration of the interactions between the full transition densities for the separate Pers, or the tetramer of Pers in a cofactor cluster with the central Chl *a* gives a different perspective. Individual Pers are found to couple with the Chl Qy state ~3–4 times stronger than the Qx state, whereas the tetramer of electronically interacting Pers couples to both Chl states to comparable extents as seen in Table 2.

Several interesting conclusions are obtained from the combined analysis of TDM re-orientation and coupling constant changes upon the introduction of Per-Per electronic interactions. (1) There is a general redistribution of coupling strength for all Per-Chl *a* pairs. Per-Qx interactions gain strength at the expenses of Per-Qy interactions. (2) Upon introducing Per-Per coupling, the TDM of the Per614-based exciton re-aligns roughly in between the polarization axes of Qx and Qy. Considering that Qx state is closest in energy to the longest wavelength absorbing Per614, this alignment and the associated coupling strength redistribution can facility energy transfer from Per614 to Chl *a*. (3) A similar argument can be applied to Per611, which is the second closest Per absorber to the Chl *a* states. Thus, Per614 and Per611 are likely the sites from which excitation energy most readily flows into the Chl *a* states. This conclusion largely confirms the energy transfer model originally proposed by Damjanovic et al. on the basis of Pariser-Parr-Pople calculations [31].

### 3.2. Environment Effect on Chromophore Coupling

We previously found that the electrostatic environment of the aqueously solvated PCP complex significantly contributes to the Per site energy regulation [8]. By varying the energy gaps between individual Pers, and/or screening inter-chromophore interactions, the electrostatic environment could, in principle, also modulate the Per-Per and Per-Chl *a* interaction strengths [32]. Alternatively, the close packing of PCP cofactors could isolate the chromophores from environmental effects [32]. We demonstrated elsewhere that static charge interactions of the Pers with the PCP environment and with one another exert opposing influences on the absorption spectrum [8]. We therefore wondered if or how the environment tunes the inter-chromophore coupling strengths.

Table 3 reports the coupling constants for the cofactor assembly of four Pers (1) isolated in a vacuum, as well as within the (2) vacuum and (3) aqueously solvated PCP complex. Chl *a* was not included in these calculations, except as part of the electrostatic environment of the PCP complex. Since intra-domain Per-Per electronic interactions have some of the strongest coupling strengths in PCP, as demonstrated below, we anticipate that any solvent-mediated effect should be maximal, and most discernable, for these interactions. The data suggests that the electrostatic PCP environment has a relatively minor (15–42 cm^−1^) influence on inter-chromophore coupling strengths. For comparison, the environment modulates the Per site energies by up to 700 cm^−1^ (17 nm) and increases the overall spectral spread of Per absorptions by a similar amount. This observation implies that the primary or most direct way in which the environment influences inter-chromophore coupling concerns the particular geometric arrangement established for the pigments in the complex. It is an intriguing observation that the four Pers of a PCP functional domain have a common directionality, with the γ-butenolide end of these chromophores oriented towards the solvent-exposed portal of the complex.

### 3.3. Relating Coupling Constants to Experiment

We present in Table 4 the inter-chromophore coupling constants greater than 100 cm^−1^. The obtained pairwise inter-pigment coupling strengths are in general agreement with previous reports [13,14,15,21,26,31]. Per-Per interactions within the same cofactor cluster tend to be the strongest, with coupling constants of 194–619 cm^−1^. Intra-cluster Per-Chl interactions are competitive with the weakest intra-domain Per-Per interactions (60–343 cm^−1^ for Pers not coupled with one another). Inter-domain Per-Per, Per-Chl, and Chl-Chl interactions tend to be <100 cm^−1^, except for a few noteworthy cases. The four possible pairwise interactions of Per612 and Per613 with Per612′ and Per613′ (where the primes indicate the C_2_-symmetry related chromophores) have strengths of 255–351 cm^−1^. Among the weakest chromophore coupling interactions, we find that the coupling constant for the Qy states of the two Chls is 15 cm^−1^, in excellent agreement with the experimental estimate of 7 cm^−1^ [33]. This observation indicates that our calculations describe inter-domain interactions remarkably well.

The finding that a few inter-domain coupling constants are competitive in strength with intra-domain chromophore interactions raises an interesting question. If chromophore coupling within a cofactor cluster significantly modulates Per site energies in order to extend the spectral range harvested by the complex, as seen in Figure 3, how do inter-domain interactions further refine the absorption spectrum?

**Figure 4:**
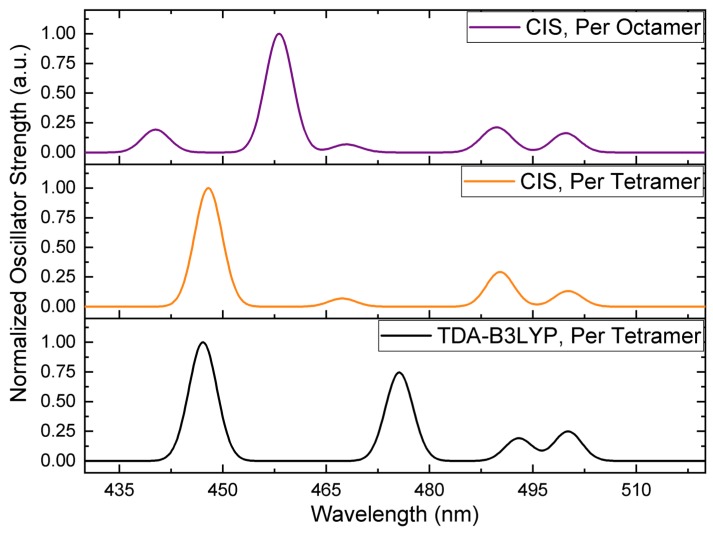
Simulated spectra for Per tetramers at the time dependent density functional theory (TD-DFT) (black) and configuration interaction with singles (CIS) (orange) levels of theory, and an octamer of Pers treated with CIS (purple), within the aqueously solvated PCP complex. All CIS wavelengths were systematically shifted by 0.671 eV to align the longest wavelength absorption with the corresponding TD-DFT band in order to aid a comparison. Gaussians with a full width at half maximum of 4 nm were fit to the calculated excitation wavelengths.

It is not computationally tractable to extend the high-level theory region of our multichromophoric QM/MM calculations over both cofactor clusters using TD-DFT. However, we were able to perform calculations with full active space configuration interaction with singles (CIS). Figure 4 compares the simulated spectra for the electronically coupled four Pers in a cofactor cluster of the solvated complex (Per tetramer) at the TD-DFT and CIS levels, and all eight Pers in the functional domains of the RFPCP homodimer (Per octamer) treated with CIS.

There are two striking features of Figure 4. First, the shortest and two longest wavelength absorption bands predicted for the Per tetramer at the TD-DFT and CIS levels are essentially identical. The most significant discrepancy in wavelength position (~10 nm) and relative intensity is found for the feature in the 460–480 nm region. The level of agreement is nonetheless acceptable.

The second important aspect of Figure 4 is that the spectrum for the Per tetramer and octamer are largely the same. However, the shortest wavelength band is split into features at shorter and longer wavelengths, thereby producing a spectrum with five Per absorptions. Interestingly, a fifth Per absorption was experimentally reported as contributing to the spectrum of the MFPCP complex [18], but the mechanistic origin of this feature, and the responsible chromophore, have yet to be identified. While it may be inferred that the electrostatic inhomogeneity of the N- and C-terminal domains of MFPCP—the two halves of the protein only share ~56% sequence identity [7]—is responsible for breaking the degeneracy of one of the symmetrically positioned pairs of Pers, our calculations show that inter-domain excitonic coupling in the absence of electrostatic differences between protein domains can produce a fifth absorption in the PCP spectrum.

One potential complication with the analysis is that the fifth absorption predicted for RFPCP was not found by spectral reconstruction for this complex [4]. However, spectral reconstruction procedures find the minimum number of absorption bands needed to approximately reproduce a spectrum, and the simulated shortest wavelength absorption for the Per octamer is not particularly intense. It is conceivable that the different electrostatic environment of MFPCP versus RFPCP works to distribute more oscillator strength into this Per S_2_ exciton. Thus, we hypothesize that the hitherto unexplained fifth short wavelength Per absorption found in the MFPCP spectrum originates from inter-domain excitonic coupling, and may gain oscillator strength under the specific electrostatic influence of the MFPCP complex. This hypothesis is in line with the finding of Carbonera et al. [13] that inter-cluster pigment interactions are necessary to accurately reproduce the circular dichroism spectrum of MFPCP. We are currently investigating our conjecture.

## 4. Conclusions

We have used supramolecular QM/MM calculations to obtain excitation energies and coupling constants with multichromophoric QM regions. Several salient aspects emerge from our calculations. We showed that the EET methodology can be used to obtain quality excitation energies for cases where the inclusion of all PCP cofactors is intractable within QM/MM at a TD-DFT level. Instead, EET can provide coupling constants which are then used to diagonalize the exciton Hamiltonian.

Intra-domain electronic coupling has a substantial effect on the S_0_ → S_2_ TDMs of each chromophore unit. Moreover, it appears that coupling aligns the TDMs of particular Per-centered excitons in a manner to favor interaction with both the Qy and Qx states of Chl *a*. We suggest that energy transfer to Chl *a* is mostly from Per614 and Per611. We also showed that the electrostatic environmental, including an implicit aqueous solvent, has a negligible effect on the strength of chromophore couplings, despite being important in the tuning of site energies. Finally, we put forward a hypothesis to explain the origin of the fifth Per absorption peak in the spectrum of MFPCP. We propose that this peak originates by the inter-domain excitonic coupling between the shortest absorber Per612 and its C_2_-pseudosymmetric counterpart.

## Figures and Tables

**Table 1:** Comparison of Per absorption wavelengths simulated with a supramolecular or fragment-based excitonic quantum mechanical (QM) approach in different environments. QM/MM: quantum mechanical/molecular mechanical; EET: electronic energy transfer.

Excitonic State	Vacuum		Solvated PCP Complex	
	Supramolecular QM/MM	EET	Supramolecular QM/MM	EET
State 1	476	478	502	513
State 2	469	468	495	501
State 3	456	458	476	482
State 4	434	434	447	454

All simulated wavelengths have been blue-shifted by 0.11 eV (~23 nm), which is the discrepancy found for the employed level of theory (Tamm-Dancoff approximation (TDA)-B3LYP/6-31g(d)) relative to experiment for Per in *n*-hexane.

**Table 2:** Comparison of Per-Chl *a* interaction strengths for uncoupled and coupled Pers in a domain of PCP.

Uncoupled Chromophores	Coupling Constants (cm^−1^)	Coupled Excitonic State	Coupling Constants (cm^−1^)
Per611-Qy	298	Per611 *-Qy	290
Per612-Qy	151	Per612 *-Qy	48
Per613-Qy	129	Per613 *-Qy	121
Per614-Qy	343	Per614 *-Qy	274
Per611-Qx	67	Per611 *-Qx	113
Per612-Qx	27	Per612 *-Qx	40
Per613-Qx	62	Per613 *-Qx	89
Per614-Qx	102	Per614 *-Qx	113

For the coupled case, an asterisk (*) indicates that the assignment is made to the chromophore with a natural transition orbital (NTO) hole-particle pair that represented at least 50% of the character of the excitation.

**Table 3:** Comparison of Per-Per interaction strengths in different environments.

Coupled Fragments	Coupling Constants (cm^−1^)		
	Vacuum	Vacuum PCP	Solvated PCP
Per611-Per612	651	609	620
Per611-Per613	311	302	278
Per611-Per614	379	372	367
Per612-Per613	372	365	353
Per612-Per614	203	200	189
Per613-Per614	473	487	468

**Table 4:** Inter-chromophore coupling constants in PCP for interactions larger than 100 cm^−1^.

Chromophore Pair	Coupling Constant	Chromophore Pair	Coupling Constant
Per611-Per612	619	Per612-Per613′	262
Per613-Per614	470	Per613-Per612′	262
Per612-Per613	377	Per613-Per613′	255
Per611-Per614	365	Per612-Per614	194
Per612-Per612′	351	Per612-Qy	151
Per614-Qy	343	Per613-Qx	129
Per611-Per613	310	Per614-Qx	102
Per611-Qy	298		
